# A comparative study to assess the work done by medical students with and without a workout routine using mosso's ergograph

**DOI:** 10.6026/9732063002002086

**Published:** 2024-12-31

**Authors:** Srivatsav B., Swathi Srinivas, Sai Sravani Sure, Suganya K., Nirangjhana S., Divyalakshmi K.

**Affiliations:** 1 Department of Medicine, Sri Ramachandra Institute of Higher Education and Research, Chennai, Tamil Nadu, India; 2 Department of Physiology, Sri Ramachandra Institute of Higher Education and Research, Chennai, Tamil Nadu, India; 3 Department of Physiology, AIIMS, Patna, India; 4 Department of Physiology, Sri Ramachandra Medical College, Porur, Chennai, Tamil Nadu, India

**Keywords:** Mosso's ergograph, medical students, work done, workout routine, sedentary lifestyle

## Abstract

A cross-sectional study was carried out to assess the differences in muscle function among healthy medical students using Mosso's ergograph. The onset of fatigue was delayed in males compared to females in both samples 1 and 2 taken with and without workout routines. The work done by males was greater than that of females in both samples 1 and 2, with and without workout routines, respectively. Physical exercise is needed to maintain a healthy weight and BMI. Many factors such as gender, frequency, duration, type of exercise and co-morbidities of the person determine the capacity and work done by that person.

## Background:

In the present scenario, a troubling paradox exists where the pursuit of academic excellence often overshadows the imperative of maintaining good health, particularly among medical students. With the advent of increasingly competitive academic environments and societal expectations for doctors, there has been a palpable shift towards prioritizing educational achievements above all else. Youngsters, driven by ambitions and societal pressures, devote substantial time and energy to academic pursuits, often at the expense of their physical and mental well-being [1]. In this milieu, health-related considerations such as regular exercise, proper nutrition and adequate rest are frequently sidelined and overshadowed by the singular focus on academic success. Neglecting physical health translates into diminished endurance, productivity and overall work capacity, compromising academic outcomes and holistic well-being [2]. Recent studies highlight the pivotal role of exercise in promoting physical well-being among students. These investigations highlight a robust correlation between physical activity and its ability to mitigate cardiovascular diseases and mental health disorders. Recommendations advocate for a structured workout routine of at least five days per week to attain optimal health and fitness [1, 2- 3]. Moreover, research indicates a positive interrelation between physical fitness and academic performance, as evidenced by heightened endurance and productivity. Numerous studies have highlighted the beneficial impact of regular exercise on cognitive function, memory retention and overall academic achievement. Moreover, individuals with higher levels of physical fitness tend to exhibit heightened endurance and productivity, enabling them to sustain focus and concentration for extended periods. This increased stamina translates into improved performance on academic tasks, including problem-solving, critical thinking and information recall [1]. However, the affordability of advanced machinery for recording work performance poses a substantial obstacle due to its prohibitive costs, impeding comprehensive physical fitness assessments among student populations. In addressing this challenge, our study employs Mosso's ergograph, a straightforward yet efficient instrument conveniently accessible within the Department of Physiology. This pragmatic strategy guarantees the acquisition of valuable insights into work capacity and endurance without incurring the burden of excessive expenditures [4]. An ergograph is a specialized instrument designed to quantitatively assess the physical work capacity of an individual by monitoring muscular contractions and energy expenditure. The instrument used for measuring and documenting the voluntary skeletal muscle contractions in humans is the ergograph where the unit of measurement of work is Erg. Mosso's ergograph is employed not only to estimate the performance of the muscles of the hand and forearm but also to study the phenomenon of fatigue and the factors affecting it [4, 5]. This study assesses physical fitness through ergographic data, to evaluate their work capacity and endurance. We aim to seek insights into the potential benefits of physical activity on the work output of medical students. This study emphasizes the importance of incorporating physical fitness into the routines of medical students as it can potentially enhance physiological capabilities and foster comprehensive student well-being.

## Materials and Methods:

## Study design:

A cross-sectional study was done to assess the effects of workout routines on work done and the onset of muscle fatigue as measured by Mosso's ergograph among medical students. This study was carried out among 114 medical students (including 57 males and 57 females) with no known co-morbidities at the Department of Physiology. A sample size of 114 was obtained by taking p=0.05 from the article: "Study of gender variation in muscle function among young adults" [1]. Institutional Ethics Committee approval (IEC approval number-CSP/23/MAY/128/403) and informed consent was obtained from all participants. A questionnaire was made to assess the background information, including age, year of study, height, weight, Body Mass Index (BMI) and details regarding the duration and frequency of workouts, which was collected from students adhering to a workout regimen. Two groups of medical students were recruited, each group consisting of 57 students. One group of students regularly performed workout routines and the other group of students did not engage regularly in any structured workout routine. Medical students above the age of 18 were included in this study and medical students with any underlying conditions were excluded. The work done by each group was evaluated using Mosso's ergograph. To initiate the assessment, the ergograph was adjusted to an appropriate height. Subsequently, the participant's forearm was secured onto the ergograph, with the index and middle fingers positioned on the designated finger holders. Notably, only the middle finger was utilized for movement during the procedure. Standard weights of 2kg and 2.5kg were used for females and 2.5kg and 3kg weights for males. Participants were instructed to pull the cord by flexing the middle finger maximally and in a rhythmic manner, with the assistance of a metronome beats application to maintain a consistent rhythm. The procedure continued until the onset of fatigue, denoted by the inability to lift the weight further. The onset of fatigue was quantified in seconds by measuring the total horizontal distance traversed until the onset of fatigue, subsequently multiplied by 2.

Work done (W) in each case was calculated using the formula:

W= F x D

Where,

W= work done (kg meters)

F= weight lifted (kg)

D= total distance moved (meters)

The value of "D" was obtained by multiplying the number of contractions with the average height of contractions.

The average height of contractions (A) was obtained by the formula,

A= Area of triangle + Area of rectangle/The total length of the base,

Therefore, W= F x D where W= F x number of contractions x A = F x number of contractions x [Area of triangle + Area of rectangle] the total length of the base

## Statistics:

The data analysis was done using Statistical Product and Service Solutions (SPSS, version 16.0; IBM SPSS Statistics for Windows, Armonk, NY). Descriptive statistics and proportions were used. Mean standard deviation and their differences were used. P<0.05 was considered as statistically significant.

## Results:

A total of 114 medical students took part in this study, including 57 male and 57 female participants respectively. The mean age of the students was 20.96 ± 1.2. The mean BMI was found to be 25.096 ± 1.9 and 25.470 ± 2.9 among the students who worked out and didn't work out respectively. A higher percentage of students maintaining a regular workout routine (63.2%) were found to have a normal BMI compared to those who did not engage in regular exercise (43.9%) (p=0.01*).

A higher percentage of students maintaining a regular workout routine (63.2%) were found to have a normal BMI compared to those who did not engage in regular exercise (43.9%) (p=0.01*) (Table 1). It was found that the percentage of students doing work out regularly is greater among males than in females (p=0.00075*). A total of 33.3% and 66.7% of females and males respectively did workout regularly whereas 64.9% and 35.1% of females and males respectively did not engage in workout. 65.7% of male participants and 42.1% of female participants reported engaging in daily workouts. Additionally, 31.57% of males and 47.36% of females reported working out approximately 4 to 5 times a week, while 2.63% of males and 10.52% of females reported exercising about 2 to 3 times a week (p=0.16) (Table 2). It was found that the onset of fatigue was delayed in males when compared to females in both students with (p=0.1) and without a workout routine (p=0.1). Males had greater work done compared to females in both the groups who worked out (p=0.1) and those who didn't work out (p=0.07). Males with a regular workout routine exhibited superior work performance compared to their counterparts who did not engage in regular exercise. Among females, those adhering to a workout routine also displayed improved outcomes compared to females without a regular workout regimen (Table 3). It was found that 42.10% of the male participants had worked out for more than two hours and 42.10% of the females had a workout routine for 1 to 2 hours a day (p=0.08) (Table 4).

## Discussion:

The study findings reveal gender disparities in physical activity levels, with a significantly higher percentage (66.7%) of male students engaging in physical activity compared to females (33.3%). Addressing these differences is crucial for developing targeted interventions to promote physical activity among both genders. This study highlights the significant impact of regular exercise on work capacity among medical students, aligning with existing literature on the correlation between endurance, physical activity and exercise. The observed increase in work done among students with a regular workout routine emphasizes the importance of incorporating regular exercise into daily routines, particularly given the demanding clinical responsibilities inherent in medical practice [6, 7]. The relationship between BMI and physical activity emerges as another critical aspect of our study. Among students with a regular workout routine, a higher proportion (63.2%) had a normal BMI compared to those leading a sedentary lifestyle (43.9%), highlighting the role of physical activity in weight management [8]. Moreover, a smaller percentage of students with a workout schedule (1.8%) fell into the obesity class I category of BMI compared to those without (5.3%), suggesting the influence of other factors such as diet and genetics. These findings highlight the importance of implementing holistic weight management strategies [9]. Over the past few decades, over-nutrition and obesity have progressed into a significant threat to public health worldwide. The Global Burden of Metabolic Risk Factors of Chronic Diseases Collaborating Group conducted an extensive analysis of data from 199 countries and territories, encompassing 9.1 million adults, to investigate the prevalence of overweight and obesity between 1980 and 2008. Over this period of 28 years, the global prevalence of obesity increased to nearly double the prevalence as previous records, with approximately 1.5 billion adults estimated to have a BMI of 25 or more by 2008 [10]. Among them, 500 million were considered obese (about 10% in men and 14% in women). In high-income countries, a negative correlation was observed between socio-economic status and obesity, majorly in women (Molarius *et al.* 2000) [11]. Whereas the prevalence of obesity was low in low and middle-income countries and confined to adults of high socioeconomic status. Monteiro *et al.* 2005 were one of the first to show that this was outdated in 2003 and that obesity had also become a problem of lower socioeconomic groups, particularly women in middle-income countries [7]. Dinsa *et al.* 2012 noted that the correlation between socioeconomic status and obesity continued to remain positive for both men and women in low-income countries [7]. The increase in obesity worldwide has a significant impact on health disorders and reduced quality of life [8]. In particular, obesity has one of the most important contributions to the global incidence of cardiovascular disease, type 2 diabetes mellitus, cancer, osteoarthritis, work disability and sleep apnea [12]. Obesity has a more pronounced effect on morbidity than on mortality. Disability due to obesity-induced type 2 diabetes would also increase, particularly in low- and middle-income countries, as the supply of insulin in these countries is comparatively insufficient [13]. Gender-based differences in the onset of fatigue were also observed, with males experiencing delayed fatigue compared to females, attributable to physiological differences such as muscle mass and hormonal variations. Furthermore, our study reveals gender-based disparities in work performance, with males exhibiting greater performance compared to females, even among those with a regular workout routine. This gender-based disparity may be influenced by a combination of physical and psychological factors [11]. Thus, our study highlights the multifaceted benefits of structured physical activity in enhancing work capacity and overall well-being among medical students. The importance of physical fitness and its impact on work capacity is highlighted, with structured physical activity corresponding to improved performance [14]. Engaging in regular exercise offers numerous benefits, including the maintenance of a healthy BMI, enhanced endurance and improved work performance. By promoting physical activity, educators and policymakers can contribute to the holistic health and professional development of future healthcare professionals [15, 16].

## Limitations:

The sample size is relatively small and this is a single-centre study. External factors and lifestyle variables like diet and sleep are not studied.

## Future recommendations:

This research underlines the need for an integrated approach in medical education, emphasizing the dual importance of physical and intellectual well-being. Further studies can be done by taking multiple other factors like diet, sleep-wake cycle, co-morbidities and study stress into consideration. Overall, this research contributes to our understanding on the numerous factors that govern physical fitness. It finally highlights the need for targeted interventions to promote physical activity and overall well-being among medical students.

## Conclusion:

In conclusion, this comparative study has shed valuable light on the relationship between workout routines and the work performance of medical students, as assessed using Mosso's ergograph. The findings emphasize the significance of regular exercise in enhancing both work capacity and endurance among medical students. This physical improvement has been shown to correlate with improved overall well-being and better work performance. It also sheds light on gender-based differences in physical activity, onset of fatigue and work performance.

## Figures and Tables

**Table 1:** Comparison of BMI among students with and without a workout routine

**Class of BMI**	**Work out**		**No work out**		**p value**
	**count**	**%**	**count**	**%**	
Normal BMI	36	63.2	25	43.9	0.01*
Overweight	20	35.1	29	50.9	
Obese class 1	1	1.8	3	5.3	

**Table 2:** Comparison of frequency of workout done among the students who exercise regularly

**Gender**	**Frequency of work out**						**p value**
	**Everyday**		**4 to 5 times a week**		**2 to 3 times a week**		
	count	%	count	%	count	%	
Males	25	65.7	12	31.57	1	2.63	
Females	8	42.1	9	47.36	2	10.52	0.16

**Table 3:** Comparison of onset of fatigue in males and females with and without a workout routine

	**Participants with a workout routine**			**Participants without a workout routine**		
	**Males**	**Females**	**p value**	**Males**	**Females**	**p value**
Onset of fatigue in sample 1 (seconds)	38	35	0.1	27	22	0.1
Onset of fatigue in sample 2 (seconds)	24	23		18	15	
Work done in sample 1 (Erg)	4	3	0.1	3	1	0.07
Work done in sample 2 (Erg)	5	4		4	2	

**Table 4:** Comparison of duration of workout among males and females

**Gender**	**Duration of work out**								**p value**
	**More than 2 hrs**		**1 to 2 hrs**		**Half an hour to 1 hr**		**Less than half an hour**		
	**count**	**%**	**count**	**%**	**count**	**%**	**count**	**%**	
Males	16	42.1	19	50	3	7.89	-	-	
Females	4	21.05	8	42.1	6	31.57	1	5.26	0.08
